# Glutathione and Its Metabolic Enzymes in Gliomal Tumor Tissue and the Peritumoral Zone at Different Degrees of Anaplasia

**DOI:** 10.3390/cimb44120439

**Published:** 2022-12-19

**Authors:** Larisa Obukhova, Tatiana Kopytova, Elena Murach, Natalya Shchelchkova, Claudia Kontorshchikova, Igor Medyanik, Natalia Orlinskaya, Artem Grishin, Michael Kontorshchikov, Dariya Badanina

**Affiliations:** Federal State Budgetary Educational Institution of Higher Education, The “Privolzhsky Research Medical University” of the Ministry of Health of the Russian Federation, 603005 Nizhny Novgorod, Russia

**Keywords:** glutathione, glutathione reductase, glutathione transferase, glutathione peroxidase, free radical activity, peritumoral zone, glial tumors

## Abstract

This research was aimed at investigating the features of free radical activity and the parameters of glutathione metabolism in tumor tissues and the peritumoral zone at different degrees of glial tumor anaplasia. We analyzed postoperative material from 20 patients with gliomas of different degrees of anaplasia. The greatest differences compared to adjacent noncancerous tissues were found in the tumor tissue: an increased amount of glutathione and glutathione-related enzymes at Grades I and II, and a decrease of these parameters at Grades III and IV. For the peritumoral zone of Grades I and II, the indices changed in different directions, while for Grades III and IV, they occurred synchronously with the tumor tissue changes. For Low Grade and High Grade gliomas, opposite trends were revealed regarding changes in the level of glutathione and the enzymes involved in its metabolism and in the free radical activity in the peritumoral zone. The content of glutathione and the enzymes involved in its metabolism decreased with the increasing degree of glioma anaplasia. In contrast, free radical activity increased. The glutathione system is an active participant in the antioxidant defense of the body and can be used to characterize the cell condition of gliomas at different stages of tumor development.

## 1. Introduction

The activation of free radical oxidation can provoke the transformation of normal cells into tumors [1]. The nervous tissue is very sensitive to oxidative stress due to the significant concentration of oxygen, the high content of polyunsaturated fatty acids in neuron membranes, and the difficulty of transporting some antioxidants through the blood-brain barrier [2]. In addition to neurotransmitters, which are capable of becoming sources of free radical compounds and activated microglia, continuously producing active forms of oxygen, the level of antioxidant protection systems in the brain is low [3,4].

An important role in the protection of cells against free radical oxidation belongs to indicators characterizing glutathione defense adequacy.

These include the concentrations of a powerful antioxidant, reduced glutathione (GSH) and its oxidized form glutathione disulfide (GSSG), plus the content and activity of glutathione peroxidase (GPx), glutathione reductase (GSR), and glutathione transferase (GST) [5]. Glutathione is an intracellular antioxidant, acting as a “trap” for free radicals as well as being a co-substrate in peroxide detoxification reactions catalyzed by glutathione peroxidase and glutathione transferase. Glutathione reductase contributes to the reduction of oxidized glutathione.

Changes in the level of reduced glutathione have been observed in various human cancer cells and are an important factor in the pathology of cancer development [6,7]. Glutathione and the enzymes involved in its metabolism participate in adaptive detoxification processes in response to oxidative stress, thus contributing to the drug resistance phenotype [7,8].

The peritumoral zone of glial tumors plays an important role in gliomagenesis. The border zone always plays a dual role, enclosing elements that, on the one hand, limit the tumor process and, on the other hand, represent a substrate for further tumor spread [9,10].

This is because the peritumoral area has a specific cellular, molecular, and biochemical composition (immune cells, various proteins, inflammatory mediators, metalloproteinases, pro- and antioxidants, different oxygen content, acidity, etc.), which distinguishes it from both tumorous and normal brain tissue. Furthermore, the parameters tend to change at different stages of the tumor process. This phenomenon can be explained by the cross-exchange of cells with adjacent zones and by changes in the biochemical parameters against the background of tumor metabolism. Processes promoting glioma progression occur in this zone. Thus, the study of the peritumoral zone is very important in order to determine the optimal resection boundaries for surgical treatment, to assess drug resistance, and to predict the likelihood of further tumor progression and recurrence [11,12,13,14].

The purpose of this study was to evaluate the characteristics of free radical activity and the parameters of glutathione metabolism in the peritumoral zone and in the tumor tissue at different degrees of glial tumor anaplasia.

## 2. Materials and Methods

### 2.1. Materials

Tissue from tumors, the peritumoral zone, and adjacent noncancerous regions were collected as postoperative material at the University Hospital of the Federal State Budgetary Educational Institution of Higher Education, the “Privolzhsky Research Medical University” of the Ministry of Health of the Russian Federation, with informed consent, before anti-tumor therapy. The exclusion criteria were as follows: under 18 years old; presence of gross somatic pathology; gliomas with multifocal growth. The histological diagnosis was made according to the WHO classification of CNS tumors [15]. Detailed information regarding the patients is listed in Additional file 1: Supplementary Table S1.

### 2.2. Preparation of Tissue Homogenate for Biochemical Research

Tissue homogenates for the biochemical studies were prepared in a refrigerated room at 0 °C. Postoperative material was washed in 0.32 M sucrose solution, pH = 7.4, and coats were removed. The tissue was then homogenized at 200 rpm in a homogenizer (glass-Teflon) in a 10-fold volume of extraction medium containing 0.32 M sucrose, 10 mM tris-HCl, and 1 mM EDTA, pH = 7.4.

### 2.3. Levels of Reactive Oxygen Species

The potential intensity of the free radical processes was measured using the BHL-07 program methodical complex of biochemiluminescence analysis (Medozons, Nizhny Novgorod, Russia). In this, the activity of free radical oxidation processes was assessed by the method of Fe-induced biochemiluminescence in terms of the intensity of the maximum flash (Imax), which reflects the potential ability of the biological object to provide free radical oxidation. The method makes it possible to register the quanta of light formed in the reactions with the forms of active oxygen. The intensity of the luminosity is affected by the full range of compounds with pro- and antioxidant action [16].

### 2.4. Analysis of the Parameters of Glutathione Metabolism

The content of reduced glutathione and glutathione disulfide in the tissue homogenates was determined by colorimetry according to the protocol of the Quantification Kit for oxidized and reduced glutathione (cat. No. 38185, Sigma-Aldrish, St. Louis, MO, USA). Absorbance was measured at 412 nm with an EPOCH tablet spectrophotometer (BIO-TEK, Winooski, VT, USA).

The amounts of each enzyme of the glutathione system in the brain tissue homogenates was studied using sandwich-type immunoassay. The determinations used the following agents: glutathione S transferase alpha 1 (GSTa1)—SEA609Hu ELISA Kit for Glutathione S Transferase Alpha 1, Cloud-Clone Corp. (PRC); glutathione reductase (GR)—SEB314Hu ELISA Kit for Glutathione Reductase, Cloud-Clone Corp. (PRC); glutathione peroxidase 1 (GPX1)—SEA295Hu ELISA Kit for Glutathione Peroxidase 1, Cloud-Clone Corp. (PRC). The optical density was recorded at 450 nm with an EPOCH tablet spectrophotometer (BIO-TEK, USA).

The amount of each enzyme was recalculated per 1 g of protein, which was determined by the Lowry method using a set of reagents from LLC “Firma Syntakon”, Russia.

### 2.5. Statistical Analysis

Statistical data were processed using the AnalystSoft Inc. package, StatPlus, version 6, Alexandria, VA, USA (www.analystsoft.com/ru/ (accessed on 28 June 2022)). The results were presented as median, quartiles of percentiles (25%; 75%). The significance of the differences was assessed using nonparametric criteria (Mann–Whitney U-criterion). Correlation was analyzed by determining the Spearman’s coefficient.

## 3. Results

Exchange Parameters of Glutathione and Reactive Oxygen Species Production Depending on the Glial Tumor Zone

The free radical activity, oxidized and reduced glutathione concentrations, plus the levels of glutathione peroxidase, glutathione reductase and glutathione transferase in the tumor tissues, the peritumoral zones, and their adjacent noncancerous tissues were evaluated (Table 1).

The free radical oxidation activity (determined by the chemiluminescence parameter Imax) was significantly reduced in the tissues of peritumoral zone of Low Grade (I, II) gliomas (Table 1). In contrast, the level of free radical oxidation in the peritumoral zones of High Grade (III, IV) gliomas was higher than in the adjacent noncancerous tissues. There was also an increase in free radical activity in the tumor tissue. Its value was significantly higher than in the tissue of the corresponding, adjacent noncancerous tissues at Grade IV (increased by 55%).

In the peritumoral tissue, there was a more than 1.5-fold decrease in the concentration of oxidized glutathione from Grades I and II to Grades III and IV. A decrease in oxidized glutathione levels was also observed in the tumor tissue. At Grade III, the amount of this compound was 2 times lower and, at Grade IV, 1.5 times lower than in the peritumoral tissue.

In Low Grade (I, II) gliomas, the content of glutathione disulfide in the peritumoral zone was significantly higher than in the adjacent noncancerous tissues, while for High Grade (III, IV) gliomas, on the contrary, it was lower. The level of reduced glutathione at Grade II was significantly greater in the tumor tissue compared to the adjacent noncancerous tissues, but a markedly lower level was observed in the High Grade (III, IV) tumors.

The level of glutathione peroxidase was increased 3-fold on average in the peritumoral area and in the tumor tissue compared to adjacent noncancerous tissues at Grade I. At Grade II, the enzyme activity was significantly increased by 3-fold in the peritumoral area (*p* = 0.004), while, in contrast, it was decreased 5-fold in the tumor tissue (*p* = 0.01). At Grade IV, an enzyme activity decrease in the adjacent noncancerous tissues to peritumoral zone tissue and further to tumor tissue were statistically significant. For Low Grade (I, II) gliomas, the glutathione peroxidase content in the peritumoral zone was significantly higher than in the adjacent noncancerous tissues, while for High Grade (III, IV) gliomas, on the contrary, it was lower. In the peritumoral tissue, there was a dramatically decreased level of the enzyme—by a factor of 5 at Grade III and by a factor of 6 in tumor tissues. In tumor tissue, there was a 5-fold decrease as early as at Grade II. The glutathione peroxidase content in noncancerous tissues did not change in respect of the degree of glioma anaplasia.

The level of glutathione reductase was significantly increased in the peritumoral region at Grade II (*p* = 0.0070), however, in tumor tissue it did not differ from the concentration of the enzyme in adjacent noncancerous tissues. At Grade III and Grade IV, there was a similar decreased glutathione reductase content in both peritumoral and tumor tissues by a factor of 3, on average. No changes of glutathione reductase levels were observed in the adjacent noncancerous tissues, whereas in the peritumoral zone the content of the enzyme was decreased, starting from Grade III, while in the tumor tissue this reduction occurred from Grade II. The trend of changes was the same in the glutathione reductase content in the peritumoral zone as for glutathione peroxidase. For Low Grade (I, II) gliomas, the concentration of glutathione reductase in the peritumoral zone was significantly higher than in the adjacent noncancerous tissues, while, in contrast, for High Grade (III, IV) gliomas it was lower.

The glutathione transferase content for Grade II gliomas was 3.5-fold higher in the peritumoral region and 2.5-fold higher in the tumor compared to adjacent noncancerous tissues. At Grade III, the level of glutathione transferase in the peritumoral tissue was significantly lower than in the adjacent noncancerous tissues. At Grade IV, a significant decrease in the concentration of glutathione transferase was characteristic only of the tumor tissue. Furthermore, an enzyme activity decrease was seen in the peritumoral zone and the tumor tissue depending on the degree of glial tumor anaplasia.

Thus, a significant result of our studies is the revealed tendency of opposite directed changes of all the investigated parameters of free radical activity compared with glutathione metabolism in the peritumoral zone in Low Grade (I, II) and High Grade (III, IV) gliomas.

## 4. Discussion

The levels of free-radical activity in the studied material changed in different ways: for Low Grade (I, II) gliomas, it was lower in the peritumoral zone than in adjacent noncancerous tissues, while for High Grade (III, IV) gliomas, it was higher. With Grade I and II, the Imax index was lower in the peritumoral zone and higher in the tumor tissue. For Grades III and IV, the index tended to be higher in both zones.

Reactive oxygen species (ROS) contribute significantly to tissue damage and tumorigenesis. They can both stimulate and inhibit carcinogenesis [17,18,19]. Activation of free radical oxidation processes can induce apoptosis [20]. Furthermore, strong oxidative stress helps to avoid this process by oxidation and inactivation of caspase enzymes [21]. Depending on the conditions, including the level of antioxidants, reactive oxygen species can either promote cell proliferation and survival [17,18] or inhibit the cell cycle [22].

Our results demonstrate a different functional role of the peritumoral zone in Low Grade and High Grade gliomas. This is consistent with the pathogenesis of gliomas, and ultimately contributes to the active growth of Low Grade tumors—a slow growth with a narrow, inactive peritumoral zone. The peritumoral zone’s oxidative processes are deactivated by high levels of glutathione and its forms. High Grade gliomas have an active peritumoral zone. The lack of glutathione or its reduced forms and of other antioxidants is accompanied by high activity of free radical processes in this zone [23].

All indicators of glutathione metabolism demonstrate the opposite trend: their levels in the peritumoral zone for Low Grade (I, II) gliomas are higher than in adjacent noncancerous tissues, while the opposite is true for High Grade (III, IV) ones.

The level of oxidized glutathione was significantly decreased compared to the adjacent noncancerous tissues both in the peritumoral zone and in the tumor tissue at all degrees of anaplasia, except for Grade II tumor tissues. The values of reduced glutathione were significantly increased compared to the adjacent noncancerous tissues only in Grade II tumor tissues and lowered both in the peritumoral zone and in Grade III and IV tumor tissues.

Glutathione is a multifunctional molecule that is a regulator of intracellular metabolism. The main role of GSH is to provide antioxidant protection of cells. In addition to its participation as a cofactor of glutathione peroxidase, GSH is capable of the non-enzymatic protection of cells from free radicals, being their trap, due to the presence of a thiol group [24]. The role of GSH in apoptosis has been established. The key mechanism in triggering apoptosis is the exit of GSH from the cells. Deprived of antioxidant protection, they die [25]. The crucial role of GSH is in the metabolism of xenobiotics and endogenous toxins [26]. It forms low-toxicity, easily excreted conjugates with drugs and other xenobiotics. Changes in intracellular GSH content are factors in the development of many pathological conditions, including carcinogenesis [27].

GSH is also considered to be the main factor responsible for treatment resistance in gliomas or other tumor cells. Thus, GSH depletion increases the susceptibility of cancer cells to various forms of programmed cell death and their sensitivity to chemotherapy [28].

As in Refs. [5,29], we have shown that changes in GSH and GSSG levels can not only be a key regulatory tool in determining the fate of cells during their differentiation into normal or tumor tissues, but it can also reflect the degree of this differentiation.

The amount of glutathione peroxidase is elevated in both tumor tissues and the peritumoral zone at Grade I, but only in the peritumoral zone at Grade II. However, it is decreased in both tissues at Grade IV and tends to be lower in both tissues in Grade III. Glutathione peroxidase is one of the most important free radical scavenging enzymes in the human body. It oxidizes GSH (reduced form) to GSSG (oxidized form), whereas H_2_O_2_ is reduced to H_2_O. Glutathione peroxidase 1 regulates the sensitivity of glioma cells to oxidative stimuli [30]. By changing the concentration of organic hydroperoxides, GPx are involved in pathways regulating cell proliferation, cell survival, and apoptosis [31].

In our study, the indicator of glutathione reductase level tends to be increased in both tumor and peritumoral tissues at Grade I. It is significantly elevated in the peritumoral zone, but in Grade II tumor tissue and in the peritumoral zone at Grade IV it does not differ from the adjacent noncancerous tissues, although it is significantly reduced in both zones at Grade III and in the tumor tissue at Grade IV. Glutathione reductase restores GSSG using NADPH_2_ as a reducing factor. Thus, the GSH redox system is linked to the balance of intracellular redox and energy metabolism through free thiol/disulfide bonds and the NADPH_2_/NADP^+^ exchange, thereby maintaining homeostasis of intracellular redox and energy metabolism.

Thus, glutathione peroxidase combined with glutathione reductase can determine not only the nature of the response to the impact of reactive oxygen species, but also the degree of drug resistance of the tumor tissue [32].

The main enzyme of glutathione metabolism, which, in addition to participation in antioxidant reactions, carries out the detoxification of electrophilic xenobiotics, is glutathione transferase (GST). A number of studies have shown that some members of the GST superfamily play a role in detoxification processes in cancers as well [33]. This enzyme is also involved in the process of the attachment of reduced glutathione to thiol groups of proteins [34]. In this way, these SH-groups of the proteins are protected from irreversible oxidation. The amount of glutathione transferase in our studies was higher within the tumors than in the adjacent noncancerous tissues at Grade I and Grade II, while it was decreased in both areas at Grade III and in tumor tissue at Grade IV, although there were no differences in levels from adjacent noncancerous tissues and the peritumoral zone at Grade IV.

Several studies have shown that altered glutathione transferase activity is relevant to multidrug resistance. Increased levels of GSTs and glutathione are closely associated with the tumor resistance to chemotherapeutic drugs [32,35]. Glutathionylation regulates the activity of the transcription factors AP1 and NF-kB, ubiquitin-dependent proteolytic protein degradation, cAMP, cAMP-dependent protein kinase and other processes. It is assumed that the increased level of reduced glutathione and GST activity is consistent with the enhancement of the S-glutathionylation processes [33,36,37]. In recent years, great regulatory importance in the process of carcinogenesis has been attributed to investigations of the Redox-sensitive signaling system represented by transcription factor Nrf2. S-glutathionylation of subunits p65 and p50 of NF-kB inhibits their binding to DNA, while S-glutathionylation of the IkB kinase β-subunit represses NF-kB activation. The Keap1/Nrf2/ARE system is traditionally considered a suppressor of tumor growth. During the development of oxidative stress, Keap1 cysteine residues are oxidized, the Keap1-Nrf2 complex dissociates, and the transcription factor translocates into the nucleus, accumulates there, and stimulates the expression of the antioxidant-responsive element genes. Recent studies have shown that GSH accumulates in the nucleus at the beginning of phase G1 [38], so it may play an important role in preserving the redox status of the nucleus during the cell cycle [39]. Thus, for NF-kB to interact with a DNA molecule, the cysteine residue in the DNA-binding domain of NF-kB should be reduced. Based on these facts and the data we have obtained, the degree of protection of proteins against proteolytic degradation at Grades III and IV is significantly reduced.

**Figure 1:**
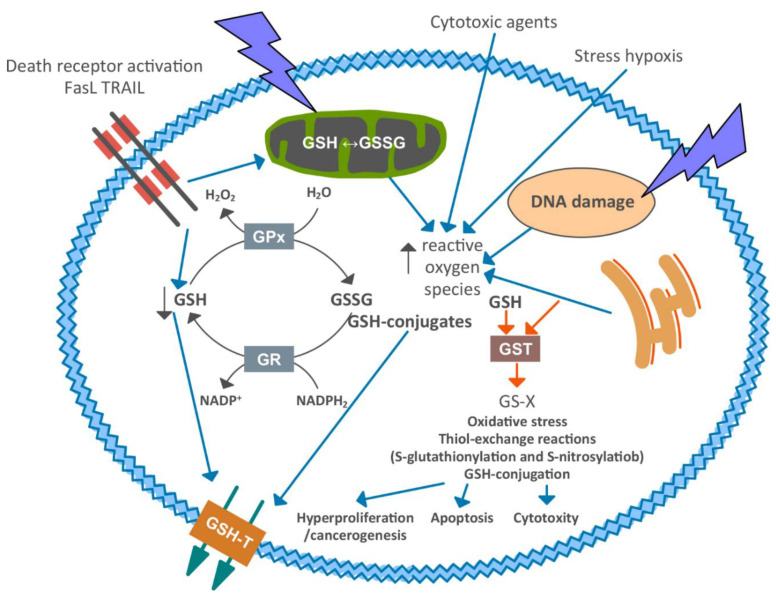
Schematic view of glutathione and its metabolism enzymes involvement in carcinogenesis [40,41] Legend: GSH—reduced glutathione; GSSG—glutathione disulfide; GPx—glutathione peroxidase; GSR—glutathione reductase; GST—glutathione transferase; GSH-T—glutathione transporter; GS-X—glutathione electrophile conjugate.

Possible involvement mechanisms of glutathione and its metabolism enzymes are shown at Figure 1.

GSH levels possibly decrease, firstly, due to the activation of its outflow from the cell membrane; secondly, due to its oxidation (GSSG formation) and/or conjugation during neutralization of reactive oxygen species formed under the influence of external stimuli and/or damage of cell organelles. Depletion of GSH and the formation of reactive oxygen species can trigger the amplification cascade. GSSG and/or GSH-conjugates are toxic to the cell and should be removed. This further depletes intracellular GSH pools by disrupting the recycling of GSSG to GSH by GR and NADPH_2_. Depletion of GSH and formation of reactive oxygen species can regulate the induction of apoptosis, cytotoxicity, or proliferative activity via thiol exchange reactions or protein oxidation modifications (S-glutathionylation, S-nitrosylation) or GSH conjugation by GST.

Summarizing our data, we can conclude that:

1. For Grade I gliomas, there is a classic picture of oxidative stress: glutathione is actively restored in response to the effects of reactive oxygen species and the levels of all the glutathione defense enzymes significantly increase;
2. At Grade II, an imbalance of the processes begins, manifested by multidirectional changes in the indicators;
3. At Grades III and IV, there is a decrease in all indicators of the glutathione pool. The amounts of reduced glutathione and glutathione disulfide decrease sharply;
4. For Low Grade (I, II) and High Grade (III, IV) gliomas, a tendency has been revealed towards opposite changes of all the examined parameters of free radical activity and of glutathione metabolism in the peritumoral zone.

Thus, the detected imbalance of the studied parameters of free radical activity and glutathione metabolism in the peritumoral zone in comparison with both the tumor tissue and the adjacent noncancerous tissues indicates the important role of this zone in tumor progression and can serve as a diagnostic and prognostic sign of the process.

## 5. Conclusions

Thus, it was observed that changes in the levels of glutathione and the enzymes involved in its metabolism, when compared with free radical activity, show opposite trends in the peritumoral zone for Low Grade and High Grade gliomas. The content of glutathione and its enzymes decreases with the increasing degree of glioma anaplasia, while free radical activity, on the contrary, increases. The glutathione system is an active participant in the body’s antioxidant defense and allows us to characterize the state of glioma cells at different stages of tumor development.

## Figures and Tables

**Table 1:** Free radical activity, glutathione levels, and glutathione metabolism enzymes in gliomas, and indicators of glutathione metabolism in gliomas.

		Adjacent Noncancerous Tissues (Median; Quartiles)	Peritumoral Zone		Tumor	
			Median; Quartiles	U-Criterion of Mann–Whitney	Median; Quartiles	U-Criterion of Mann–Whitney
Free radical activity (I max), mV	Grade I (*n* = 1)	121	97		169	
	Grade II, (*n* = 6)	265.50 (264.00–290.25)	173.00 (155.75–190.25)	* *p* = 0.004	227.50 (209.25–341.75	*p* = 0.393
	Grade III, (*n* = 3)	130.00 (130.00–180.50)	205.00 (187.50–218.00)	*p* = 0.275	215.00 (189.50–273.00)	*p* = 0.275
	Grade IV, (*n* = 10)	230.50 (163.25–293.25)	274.00 (221.50–327.25)	*p* = 0.200	357.60 (334.750–381.00)	* *p* = 0.011
Oxidized glutathione, μmol/L	Grade I (*n* = 1)	12,583.00	13,636.00		10,532.00	
	Grade I, (*n* = 6)	11,514.50 (10,897.465–12,141.75)	14,359.00 (13,350.75–15,753.50)	* *p* = 0.054	11,430.50 (9772.00–12,059.25)	*p* = 0.631
	Grade III, (*n* = 3)	12,256.00 (11,191.00–12,661.04)	8137.00 (8124.79–9019.50)	* *p* = 0.049	4013.00 (3635.89–4975.50)	* *p* = 0.050
	Grade IV, (*n* = 10)	11,363.33 (11,146.00–11,853.17)	9021.00 (8462.97–9837.00)	* *p* = 0.007	6854.65 (6464.75–7006.58)	* *p* = 0.004
Reduced glutathione, μmol/L	Grade I (*n* = 1)	11,239.00	15,210.00		1323.00	
	Grade II, (*n* = 6)	9342.00 (8783.75–9922.75)	10,639.50 (7043.55–11,928.00)	*p* = 0.522	14,132.00 (13,294.81–14,675.25)	* *p* = 0.003
	Grade III, (*n* = 3)	10,396.00 (10,033.00–11,347.78)	6921.00 (5174.08–7725.50)	* *p* = 0.049	7813.00 (7059.00–8641.78)	* *p* = 0.049
	Grade IV, (*n* = 10)	9705.62 (9417.06–9970.00)	8004.50 (6478.75–8315.22)	*p* = 0.109	8006.50 (7777.00–8636.50)	* *p* = 0.054
Glutathione peroxidase, ng/g of protein	Grade I (*n* = 1)	8.25	24.71		21.30	
	Grade II, (*n* = 6)	6.84 (5.89–7.44)	20.00 (19.25–21.50)	* *p* = 0.004	3.79 (3.52–4.73)	* *p* = 0.010
	Grade III, (*n* = 3)	6.43 (4.33–7.06)	3.88 (2.89–4.07)	*p* = 0.275	3.78 (2.43–5.12)	*p* = 0.513
	Grade IV, (*n* = 10)	7.72 (7.06–12.89)	3.22 (2.56–4.37)	* *p* = 0.039	2.63 (1.41–3.78)	* *p* = 0.0002
Glutathione reductase, pg/g of protein	Grade I (*n* = 1)	2105.00	3179.00		2769.23	
	Grade II, (*n* = 6)	1802.00 (1625.25–1872.17)	3188.50 (3029.00–3452.25)	* *p* = 0.007	1536.49 (1351.56–1698.41)	*p* = 0.149
	Grade III, (*n* = 3)	1750.28 (1657.14–1810.64	973.00 (569.84–987.00)	* *p* = 0.049	1134.00 (895.02–1338.36)	* *p* = 0.049
	Grade IV, (*n* = 10)	1894.00 (1482.49–2252.00)	994.35 (685.42–1739.20)	*p* = 0.065	907.79 (420.39–1374.25)	* *p* = 0.011
Glutathione transferase, pg/g of protein	Grade I (*n* = 1)	28.25	78.19		67.31	
	Grade II, (*n* = 6)	20.07 (16.60–22.08)	70.70 (66.60–74.61)	* *p* = 0.004	54.47 (41.82–61.49)	* *p* = 0.007
	Grade III, (*n* = 3)	27.00 (26.02–28.50)	19.00 (18.52–21.00)	* *p* = 0.005	15.00 (13.48–24.17)	*p* = 0.513
	Grade IV, (*n* = 10)	35.17 (32.89–55.20)	33.24 (23.21–40.51)	*p* = 0.064	12.09 (7.37–16.32)	* *p* = 0.001

Legend: * statistically significant differences in comparison with the adjacent noncancerous tissues of the brain.

## Data Availability

The datasets generated during and/or analyzed during the current study are available from the corresponding author on reasonable request.

## Supplementary Materials

The following supporting information can be downloaded at: https://www.mdpi.com/article/10.3390/cimb44120439/s1, Table S1: Clinicopathologic features of gliomas patients.
