# Supplementary material for: Glutathione and Its Metabolic Enzymes in Gliomal Tumor Tissue and the Peritumoral Zone at Different Degrees of Anaplasia
Source: Curr Issues Mol Biol. 2022 Dec 19;44(12):6439–49. doi: 10.3390/cimb44120439 (PMC9777065; doi:10.3390/cimb44120439)
Supplement: Supplementary file 1 [file cimb-44-00439-s001.zip › cimb-2049887-supplementary.pdf]

Supplementary information

Additional file S1 of « RELATIONSHIP OF GLUTATHIONE-DEPENDENT ENZYMES AND THE IMMUNOHISTOCHEMICAL PROFILE OF GLIAL NEOPLASMS»

Supplementary Table S1. Clinicopathologic features of gliomas patients

Characteristics	Grade I (n=1)	Grade II (n=6)	Grade III (n=3)	Grade IV (n=10)
Ages (years)				
< 60	1	2	2	3
≥ 60		4	1	7
Gender				
Male		2	2	7
Female	1	4	1	3
Average tumor volume (cm/^{3})	121,9	99,06	66.6	89,7

Within the framework of the studied material, pilocytic astrocytoma was classified as Grade I. Two 1p/19q-codeleted oligodendrogliomas and four IDH-mutant astrocytomas were included into Grade II. Group Grade III included two 1p/19q-codeleted oligodendrogliomas and one IDH-mutant astrocytoma. Group Grade IV included six IDH-wild-type glioblastomas, three IDH-mutant astrocytomas, and one H3K27m midline glioma.
